# MSC Secretome as a Promising Tool for Neuroprotection and Neuroregeneration in a Model of Intracerebral Hemorrhage

**DOI:** 10.3390/pharmaceutics13122031

**Published:** 2021-11-29

**Authors:** Maxim Karagyaur, Stalik Dzhauari, Nataliya Basalova, Natalia Aleksandrushkina, Georgy Sagaradze, Natalia Danilova, Pavel Malkov, Vladimir Popov, Mariya Skryabina, Anastasia Efimenko, Vsevolod Tkachuk

**Affiliations:** 1 Institute for Regenerative Medicine, Medical Research and Education Center, Lomonosov Moscow State University, 27/10 Lomonosovsky Ave, 119192 Moscow, Russia; natalia_ba@mail.ru (N.B.); n.alexandrushkina@mc.msu.ru (N.A.); gsagaradze@mc.msu.ru (G.S.); galiantus@gmail.com (V.P.); aefimenko@mc.msu.ru (A.E.); tkachuk@fbm.msu.ru (V.T.); 2 Faculty of Medicine, Lomonosov Moscow State University, 27/1 Lomonosovsky Ave, 119192 Moscow, Russia; stalik.djauari@yandex.ru (S.D.); ndanilova@mc.msu.ru (N.D.); pmalkov@mc.msu.ru (P.M.); skrebbka@gmail.com (M.S.)

**Keywords:** multipotent mesenchymal stromal cells (MSC), secretome, intracerebral hemorrhage, stroke, brain-derived neurotrophic factor, allogenicity, glutamate-induced neurotoxicity, microglia activation

## Abstract

Multipotent mesenchymal stromal cells (MSCs) are considered to be critical contributors to injured tissue repair and regeneration, and MSC-based therapeutic approaches have been applied to many peripheral and central neurologic disorders. It has been demonstrated that the beneficial effects of MSC are mainly mediated by the components of their secretome. In the current study, we have explored the neuroprotective potential of the MSC secretome in a rat model of intracerebral hemorrhage and shown that a 10-fold concentrated secretome of human MSC and its combination with the brain-derived neurotrophic factor (BDNF) provided a better survival and neurological outcome of rats within 14 days of intracerebral hemorrhage compared to the negative (non-treated) and positive (BDNF) control groups. We found that it was due to the ability of MSC secretome to stimulate neuron survival under conditions of glutamate-induced neurotoxicity. However, the lesion volume did not shrink in these rats, and this also correlated with prominent microglia activation. We hypothesize that this could be caused by the species-specificity of the used MSC secretome and provide evidence to confirm this. Thus, we have found that allogenic rat MSC secretome was more effective than xenogenic human MSC secretome in the rat intracerebral hemorrhage model: it reduced the volume of the lesion and promoted excellent survival and neurological outcome of the treated rats.

## 1. Introduction

Multipotent mesenchymal stromal cells (MSCs) were initially identified by Friedenstein et al. and defined as plastic-adherent fibroblast colony-forming units with clonogenic capacity [1]. Later, MSCs were found in the perivascular niche and interstitial compartment of a wide variety of tissues [2]. They are considered as critical contributors to tissue-specific stem cell niche maintenance and restoration after damage [3]. It has previously been shown that MSCs are also able to regulate neural progenitor cell proliferation and differentiation found in the neurogenic niche [4]. Multiple studies have demonstrated the direct antiapoptotic activity of MSCs as well as their ability to stimulate angiogenesis and neurogenesis, and to suppress the immune response [5,6,7,8,9]. This variety of pro-regenerative activities makes MSCs a perspective tool for clinical therapy [10].

An accumulating amount of experimental data indicates that the most beneficial and protective effects of MSCs are mediated by the secretion of biomolecules and soluble factors, including many growth factors, cytokines, and noncoding RNAs, as well as the transfer of organelles and extracellular vesicles to target cells, and producing the extracellular matrix component, which has structural and signaling functions [3,11,12]. Many of these factors have been reported to possess protective effects on the nervous tissue [5,13].

However, the potential mechanisms of the beneficial role of MSCs in the central nervous system recovery following damage mediated by their secretome are still poorly understood. We think that an experimental model of intracerebral hematoma may be helpful to clarify this, since spilled blood triggers a wide range of pathological processes within the brain tissue (direct neurotoxicity, damage to nerve fibers, inflammation, ischemia, fibrosis, etc.), which MSCs are able to modulate [14,15]. Here, we have tried to ascertain the neuroprotective and pro-regenerative contribution of MSC secretome components in the restoration of the rat brain in the intracerebral hemorrhage model.

## 2. Materials and Methods

### 2.1. Animals

The mature male Wistar rats used in this study were between 3.0 and 3.5 months old and had standard weight characteristics. Animals were housed and used for experimental procedures in full compliance with Directive 2010/63/EU and the recommendations of the Bioethics Committee of Lomonosov MSU (permission #3.4, 03/21/2021).

### 2.2. Cell Culture

Human MSCs derived from adipose tissue of healthy donors (n = 3) were obtained from the biobank of the Institute for Regenerative Medicine, Lomonosov MSU, collection ID: MSU_MSC_AD (https://human.depo.msu.ru), and cultured in a medium supporting the growth of undifferentiated mesenchymal progenitor cells (Advance Stem Cell Basal Medium, HyClone, South Logan, UT, USA) containing 10% of a growth factor supplement (Advance Stem Cell Growth Supplement, HyClone, South Logan, UT, USA) and 100 U/mL of penicillin/streptomycin (Gibco). All procedures performed with tissue samples from patients were in accordance with the Declaration of Helsinki and approved by the Ethic Committee of Lomonosov Moscow State University (IRB00010587), protocol #4 (2018).

Rat MSCs derived from adipose tissue were obtained from mature male Wistar rats (see Section 2.1) according to the previously described protocol [16] and cultured in Dulbecco’s Modified Eagle Medium (DMEM) supplemented with 10% fetal bovine serum (FBS) and 100 U/mL of penicillin/streptomycin (all from Gibco, Grand Island, NY, USA).

Obtained MSCs were characterized as being plastic adherent, expressing CD73, CD90, and CD105, lacking the expression of hematopoietic and endothelial markers CD14, CD19, CD34, CD45, and HLA-DR, and capable of in vitro differentiation into adipocyte, chondrocyte, and osteoblast lineages, which met the criteria set by the International Society for Cellular Therapy (ISCT) [17,18]. The medium was changed every three to four days. All experiments were performed with cells within five passages.

SH-SY5Y cells were obtained from ATCC (CRL-2266™) and cultured in DMEM/F12 medium supplemented with 10% FBS and 1% penicillin/streptomycin (all from Gibco, Grand Island, NY, USA). The medium was changed every three to four days.

### 2.3. Manufacturing of Cell Secretomes

MSC-conditioned medium contained components of human or rat MSC secretome was obtained according to the previously published protocol [19]. Briefly, subconfluent human or rat MSCs at passages four to five were thoroughly washed with Hanks solution (PanEko, Moscow, Russia) and were then cultured for seven days in DMEM containing low glucose (DMEM-LG), GlutaMAX™ Supplement, pyruvate and 100-U/mL penicillin/streptomycin (all from Gibco, Grand Island, NY, USA). The medium of human MSCs was then removed and centrifuged for 10 min at 300× *g* to remove cell debris (hMSC1x group), and a part of the sample was concentrated tenfold (hMSC10x group) using a centrifugal ultra-filter with 10-kDa molecular weight cut off (MWCO; Merck, Tullagreen, Ireland). The rat secretome was concentrated 10 fold in the same way (rMSC10x group). 10-fold concentration was used due to our previous experiments on in vitro models, demonstrating that the efficiency of MSC secretome to impact cell behavior reached its plateau when MSC secretome was 7,5-10-fold concentrated.

As a negative control (Control group), we used DMEM-LG supplemented with GlutaMAX™ Supplement, pyruvate (all from Gibco, Grand Island, NY, USA), and 100 U/mL penicillin/streptomycin. As a positive control, we used the same control medium supplemented with 3.5 ng/μL of human brain-derived neurotrophic factor (BDNF) (#ab206642-2, Abcam, Waltham, MA, USA) (BDNF group) [20]. To increase the neuroprotective activity, the hMSC1x secretome was supplemented with human BDNF to a final concentration of 3.5 ng/μL (MSC-B group).

### 2.4. Intracerebral Hemorrhage Modelling and Secretome Administration

The study was carried out on a model of intracerebral hemorrhage into the inner capsule of the right hemisphere, according to A.N. Makarenko et al. [21]. The inner capsule contains many neural tracts that connect the cortex to the basal ganglia, nuclei of the spinal cord, and cranial nerves. Therefore, injury to the inner capsule causes various neurological deficits, and their severity correlates with brain injury severity.

The study included 98 animals: 40 rats for the pilot study (sham operated (SO), Control, BDNF, and hMSC1x groups, 10 rats each), 30 rats for the study of the neuroprotective activity of the human MSC secretome (Control, hMSC10x, and MSC-B groups, 10 rats each), and 28 rats for the species-specificity study (10 rats for the Control group and 18 for the rMSC10x group). None of the rats were excluded from the study. To model the hemorrhage, rats were anesthetized with a solution containing 2% Zoletil^®^ 50 (VirBac, Carros Cedes, France) and 1.5% xylazine (InterChemie, Venray, the Netherlands) in saline, at a dose of 1 mL/kg body weight and positioned in a stereotaxic frame. The scalp and skull aponeuroses were dissected, and the cranium was perforated (bregma −2.0 mm, lateral 3.5 mm) [22]. Local brain tissue was destroyed, and the superior cerebral veins on the corresponding side were injured to model a bleed. To standardize the volume of spilled blood, 20 uL of autologous blood obtained from the hypoglossal vein were slowly injected into the injury focus. In 5 min, the blinded investigator injected 20 uL of the appropriate concentrated medium sample and the wound was sutured. The animals from the SO group underwent cranium perforation without any subsequent brain damage or blood/medium sample injections.

### 2.5. Neurological Status Assessment

The animals were observed for 14 days following the intracerebral hemorrhage, with daily recording of rat deaths and a neurological status assessment at three and 10 days following the hemorrhage (Stroke-index McGraw scale, modified for rodents by I.V. Ganushkina) [23,24]. Briefly, “visually healthy animals” had no signs of neurological deficit; animals with signs of lethargy, limb weakness, tremor, ptosis, and/or semi-ptosis were considered “slightly affected”; and animals demonstrating signs of paresis and/or paralysis of the limbs, impaired coordination, or in a coma were considered “severely affected”. The researchers conducting the neurological testing were blinded to each rat’s treatment.

Cognitive impairment was assessed using the passive avoidance test [25]. The animals were trained for the reflex 24 h before the intracerebral hemorrhage. For this, each animal was placed in the passive avoidance apparatus (San Diego Instruments, San Diego, CA, USA), and the latent time prior to its entry into the dark chamber of the apparatus was recorded. In the chamber, the rat received eight painful electric stimuli (0.4 mA, 1 s each). The animals were then re-examined 24 h and 10 days after the intracerebral hemorrhage (short-term and long-term memory, respectively) and the time of avoidance of the dark chamber with the electrical floor was recorded. The maximum observation time for each animal was 180 s. The latent time results were normalized to 180 s and the efficacy of short-term and long-term memory were obtained as a percentage.

### 2.6. MRI

MRI were obtained 11 days following the intracerebral hemorrhage using the Clinscan 7T system (Bruker Biospin, Billerica, MA, USA) equipped with the rat brain surface coil with TurboS Spin Echo sequence and fat suppression. The coronary projections were acquired using the following parameters: TR (Repetition Time) = 5220 ms; TE (Time to Echo) = 53 ms; echo train length = 9; base resolution 230 × 320; FoV (Field-of-View) = 32 × 40 mm; slice thickness = 0.5 mm; spacing between the slices = 0.75 mm. The transversal projections were acquired using the following parameters: TR = 4000 ms; TE = 40 ms; echo train length = 9; base resolution 288 × 320; FoV = 40 × 40 mm; slice thickness = 0.5 mm; spacing between the slices = 0.6 mm.

### 2.7. Histochemistry and Immunohistochemistry

For histological studies, the rats were euthanized on day 14 following the intracerebral hemorrhage by exposure to gradually increasing concentrations of CO_2_. The brain was removed, fixed in 4% formaldehyde solution, and embedded in paraffin. The brain slices containing the injury focus were dewaxed, and antigens were unmasked. Some of the slices were stained with hematoxylin-eosin, cresyl violet (Nissl stain), or Perls Prussian blue (Perls stain). A combination of these stains allowed assessment of the following aspects of morphological changes within the injured brain: the size of the lesion, neuronal status, leukocyte infiltration rate, and the size of hemosiderin deposits.

For immunohistochemical staining, the slices were treated with 50 mM ammonium acetate solution and blocked with 10% goat serum to reduce background fluorescence. After blocking, they were incubated with antibodies to CD68 (Abcam, #ab125212, Waltham, MA, USA) and CD163 (Abcam, #ab182422, Waltham, MA, USA), followed by incubation with fluorescently-labeled secondary goat-anti-rabbit antibodies (Invitrogen, #A11034, Waltham, MA, USA). The nuclei were stained with 4′,6-diamidino-2-phenylindole (DAPI) solution (Sigma, #MBD0015-10ML, St. Louis, MO, USA). The specimens were examined with a Leica DM600 microscope equipped with a DFC360 FX and DFC420C camera (Leica Microsystems GmbH, Wetzlar, Germany), using representative fields of view to obtain the photographs. Image processing and analysis were performed using LasX software (Leica Microsystems GmbH, Wetzlar, Germany) and FiJi.

### 2.8. In Vitro Study of Cell Secretome Neurotrophic Activity

To assess the direct neuroprotective activity of MSC secretomes, we used a previously published in vitro model of glutamate-induced neurotoxicity [26,27]. This cellular model reflects one of the main causes of neuronal death during stroke: glutamate-induced neurotoxicity. For this endeavor, SH-SY5Y neuroblastoma cells were seeded in 48-well plates in complete growth medium at 40,000 cells/well in quadruplicates. The medium was removed the next day, and samples of serum-free medium (C+), serum-free medium with 3.5 ng/μL human BDNF (BDNF) or MSC secretome (hMSC1x, hMSC10x, MSC-B, or rMSC10x), were added, each supplemented with 100 mM L-glutamate [26] and 5 μM IncuCyte^®^ Caspase-3/7 Apoptosis Reagent (Essen Bioscience, #4440, Ann Arbor, MI, USA). In the control group C-, no L-glutamate, BDNF, or MSC secretome were added to monitor the spontaneous cell death in serum-free medium. Glutamate causes a rapid increase in the cytosolic concentration of Ca^2+^ in SH-SY5Y cells (similar to what happens in neurons), with subsequent caspase activation. The IncuCyte^®^ Caspase-3/7 Apoptosis Reagent, cleaved with activated caspase-3/7, stains nuclear DNA (green fluorescence). To monitor the death of SH-SY5Y cells, the plate was placed in the Incucyte^®^ ZOOM Live Cell Analysis System (Essen Bioscience, Ann Arbor, MI, USA), located inside a carbon dioxide (CO_2_) incubator. The time-lapse imaging of nine fields of vision (phase and green channel) of each well was performed 1, 3, 6, 12, 24, 48, and 72 h after medium replacement.

To assess the ability of MSC secretomes to stimulate neurite outgrowth, SH-SY5Y cells were seeded in 48-well plates in complete growth medium at 40,000 cells/well in quadruplicates. The medium was removed the next day, and serum-free medium (Control), serum-free medium with 3.5 ng/μL of human BDNF (BDNF), or MSC secretome (hMSC1x, hMSC10x, MSC-B, or rMSC10x) were added. To monitor the neurite outgrowth of SH-SY5Y cells, the plate was placed in the Incucyte^®^ ZOOM Live Cell Analysis System (Essen Bioscience, Ann Arbor, MI, USA), located inside a CO_2_ incubator. The time-lapse imaging of nine fields of vision of each well was performed 0, 6, and 24 h after medium replacement.

The micrographs were analyzed with ImageJ (version 1.41) software (National Institutes of Health, Bethesda, MD, USA) to calculate the percentage of living cells (compared with the initial cell number) and neurite outgrowth at each time point. Cell processes longer than two cell diameters were considered neurites.

### 2.9. Statistical Analysis

Statistical analysis was performed using SigmaPlot11.0 software (Systat Software, Inc., Erkrath, Germany). Numerical data was assessed for normality of distribution using the Kolmogorov–Smirnov test. Differences between the treatment and control groups were analyzed using Student’s *t*-test or analysis of variance (ANOVA) on ranks (Dunn’s test), depending on whether the data were normally distributed. Data are expressed as the mean ± standard deviation or the median (25%; 75%), depending on the test used. We considered differences to be significant when *p* < 0.05.

To analyze the categorical data (neurological outcome after stroke), Fisher’s exact test was used: BDNF, hMSC1x, hMSC10x, MSC-B, and rMSC10x groups were compared pairwise to the control group. Since Fisher’s exact test only supports 2 × 2 massives (χ2 is not applicable for small groups), the proportions of animals were combined into two groups: “visually healthy animals” + “slightly affected”, “severely affected” + “dead”. Different points in time were compared separately.

## 3. Results

### 3.1. The Secretome of Human MSCs Protects the Brain after the Intracerebral Hemorrhage

**Figure 1:**
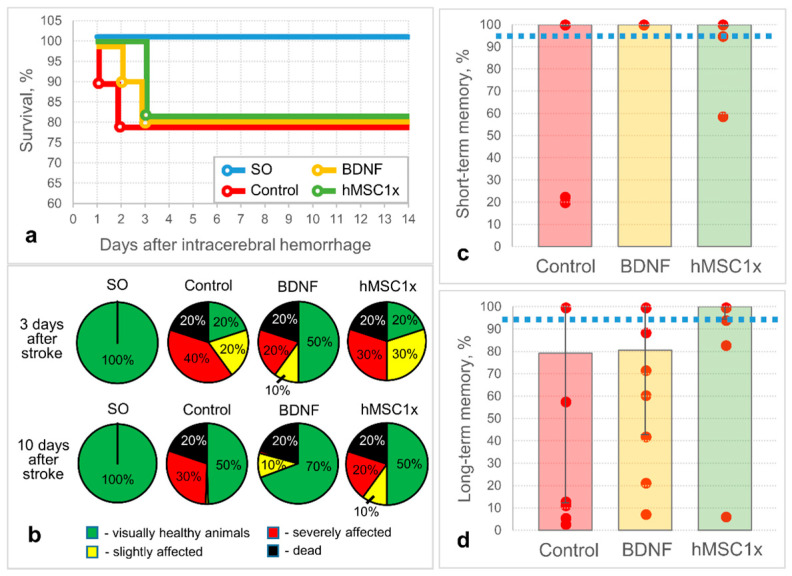
The results of in vivo studies of the neuroprotective activity of the non-concentrated human MSC secretome (hMSC1x): (**a**) survival of the experimental animals; (**b**) neurological status of the experimental animals (SO—sham operated); and the cognitive status of the experimental animals: (**c**) short-term memory (24 h after the intracerebral hemorrhage); (**d**) long-term memory (10 days after the intracerebral hemorrhage). The dashed line corresponds to the memory performance of SO animals. Data is presented as a median (25%; 75%). n = 10; no significant differences between the groups.

Here, we studied the protective and pro-regenerative properties of human MSC secretome in a rat model of intracerebral hemorrhage. The results of the pilot study showed that hMSC1x secretome has potential therapeutic activity, but it turns out to be less effective than the positive control BDNF secretome (Figure 1b). Although hMSC1x secretome did not have a significant effect on survival (Figure 1a) and the alleviation of neurological deficits in experimental animals, it contributed to the preservation of long-term memory in rats [100% (94.4%; 100%) versus 79.2% (12.2%; 100%) in the control group, n ≥ 8] (Figure 1d), whereas BDNF provided a better neurological outcome (Figure 1b), but was less effective than hMSC1x in the cognitive test: 80.6% (42.8%; 100%) (Figure 1d). Presumably, this is due to the difference in action of BDNF and hMSC1x secretome: BDNF is a protein with direct (immediate) neurotrophic activity, while hMSC1x secretome consists of many molecular complexes (especially microvesicles that have a delayed effect on the transciption program of the target cells) with a lower content of neurotrophic factors (e.g., BDNF concentration 3 ± 1.2 pg/μL, n = 9). The short-term memory test revealed no difference between the groups (Figure 1c).

We hypothesized that the weak effect of the hMSC1x secretome is due to the low concentration of biologically active components within the secretome, so we conducted an extended study using the combination of hMSC1x complemented with human BDNF 3.5 ng/μL (MSC-B) and 10-fold concentrated human MSC secretome (hMSC10x). The addition of the MSC-B group to the experiment was due to the expectation of an additive therapeutic effect of the BDNF protein (immediate) and MSC microvesicles (delayed effect) during the long brain recovery process after the intracerebral hemorrhage.

**Figure 2:**
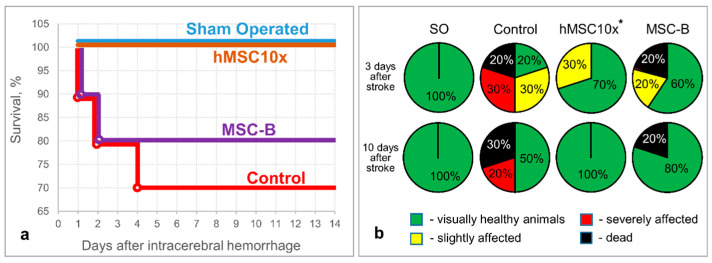
The results of in vivo studies of the neuroprotective activity of the 10 fold-concentrated human MSC secretome (hMSC10x): (**a**) survival of the experimental animals; (**b**) neurological status of the experimental animals at three and 10 days after the intracerebral hemorrhage (SO—sham operated). Data is presented as a median (25%; 75%). [*—*p* = 0.006; n ≥ 10; hMSC10x vs. Control; two-sided Fisher’s exact test].

The hMSC10x secretome supported neurological health (at 10 days after the intracerebral hemorrhage) and provided the survival of 100% of experimental animals (up to 14 days after the intracerebral hemorrhage), while 30% of animals died and 20% of animals had a severe neurological deficit in the negative control group (Control) (at 10 days after the intracerebral hemorrhage) [*p* = 0.006; n ≥ 10; two-sided Fisher’s exact test] (Figure 2). A decrease in the severity of neurological deficits, without a significant increase in survival (80%), was observed in the MSC-B experimental group (as well as in the BDNF and hMSC1x groups).

**Figure 3:**
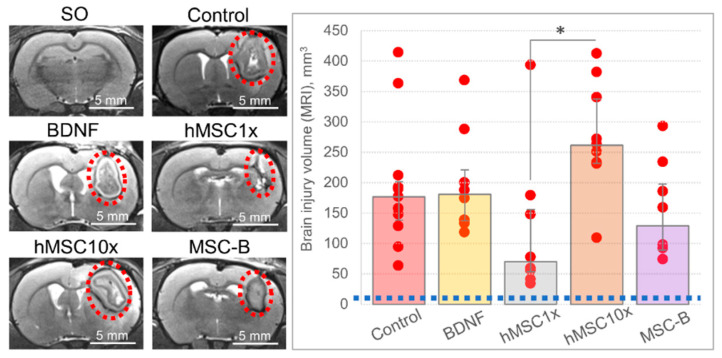
The results of the MRI examination of the brain at day 11 after the intracerebral hemorrhage. On the diagram: the blue dashed line equals the volume of the brain lesion site in the SO animals. Data is presented as a median (25%; 75%). [*—H (4,35) = 11.841; *p* = 0.019; n ≥ 8; ANOVA on ranks].

According to the MRI study, there were no significant differences in the brain lesion volume in the groups compared to the control. A tendency to diminution was observed in the hMSC1x group, while the hMSC10x group showed a tendency for the brain lesion volume to increment at 11 days after the intracerebral hemorrhage: 70.1 (53.5; 155.8) mm^3^ and 261.4 (232.0; 337.4) mm^3^, respectively, versus 176.6 (138.5; 201.5) mm^3^ in the control group (Figure 3). However, a significant difference in the lesion volume size was observed between the hMSC1x and hMSC10x groups [H (4,35) = 11.841; *p* = 0.019; n ≥ 8; ANOVA on ranks].

**Figure 4:**
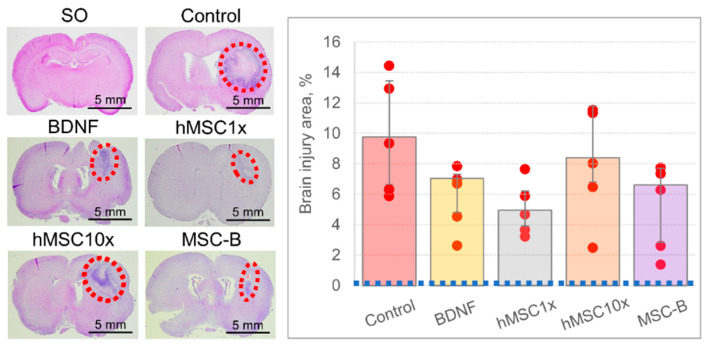
The results of the histochemical examination of the brain at day 14 after the intracerebral hemorrhage. On the photos: the red dashed ovals restrict the injured loci. On the diagram: the blue dashed line equals the area of the brain lesion site in the SO animals. Data is presented as a median (25%; 75%). n = 5; no significant differences between the groups.

Hematoxylin-eosin staining of the brain slices across the injury site revealed the similar distribution of the brain lesion size across the groups (Figure 4). No significant difference was observed between the groups, although as in the MRI study, the lesion site had a tendency to diminution in the hMSC1x and MSC-B groups: 4.9% (3.9%; 6.2%) and 6.6% (2.8%; 7.7%), respectively, versus 9.8% (6.6%; 13.4%) in the control group [H (4,20) = 5.738; *p* = 0.220; n = 5; ANOVA on ranks].

Hematoxylin-eosin and Nissl staining of the brain slices also confirmed the presence of microscopic signs of brain tissue damage: necrosis, ischemia, edema, glial scar, neutrophilic infiltration, and blood stasis in the capillary bed (Figures S1 and S2). The signs of damage were more prominent in the control group, but quantitative analysis was not performed. Perls Prussian blue staining demonstrated deposits of hemosiderin (blue) in the brain tissue adjacent to the lesion site in all groups, but were less expressed in the Control and hMSC10x groups (Figure S2). We believe that this demonstrates that the expansion of the primary lesion site engulfed hemosiderin deposites in the Control and hMSC10x groups.

**Figure 5:**
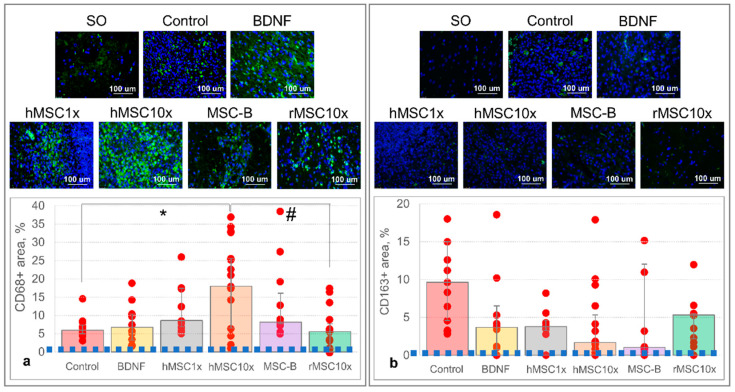
The results of the immunohistochemical staining of brain slices at day 14 after the intracerebral hemorrhage: (**a**) immunostaining for CD68; (**b**) immunostaining for CD163. On the photos: the green staining corresponds to the CD68 or CD163 staining; the blue staining corresponds to cell nuclei. On the diagram: the blue dashed line equals the area of the brain lesion site in the SO animals. Data is presented as a median (25%; 75%). [*—H (4,40) = 10.404, *p* = 0.034, n ≥ 9, ANOVA on ranks; #—H (5,48) = 16.689, *p* = 0.005, n ≥ 9, ANOVA on ranks].

Immunohistochemical staining of brain slices for CD68, a proinflammatory M1-microglia cell and lysosomal activity marker, revealed its significantly higher representation in the brain sections of the hMSC10x group: CD68-specifically stained area—18.0% (6.5%; 25.6%) versus 5.9% (5.0%; 7.4%) in the control group [H (4,40) = 10.404; *p* = 0.034; n ≥ 9; ANOVA on ranks] (Figure 5a). No significant difference was observed in the CD68-stained area in the BDNF, hMSC1x, and MSC-B groups compared to the control group (Control), although there was a tendency towards an increase in the number of CD68+cells in these groups. rMSC10x secretome effect will be described in the Section 3.2.

Staining for CD163, a marker of M2-microglia cells, showed a prominent (but not statistically significant) decrease in the level of CD163+cells in the hMSC10x group—1.7% (0.6%; 5.3%) compared to 9.7% (4.6%; 15.0%) in the control group [H (4,35) = 9.410; *p* = 0.052; n ≥ 8; ANOVA on ranks] (Figure 5b). A similar decrease was observed in the BDNF, hMSC1x, and MSC-B groups, although they still did not differ significantly from the control group. rMSC10x secretome effect will be described in the Section 3.2.

### 3.2. An Allogenic MSC Secretome Is as Effective and Safer Than a Xenogenic MSC Secretome for Brain Recovery after the Intracerebral Hemorrhage

**Figure 6:**
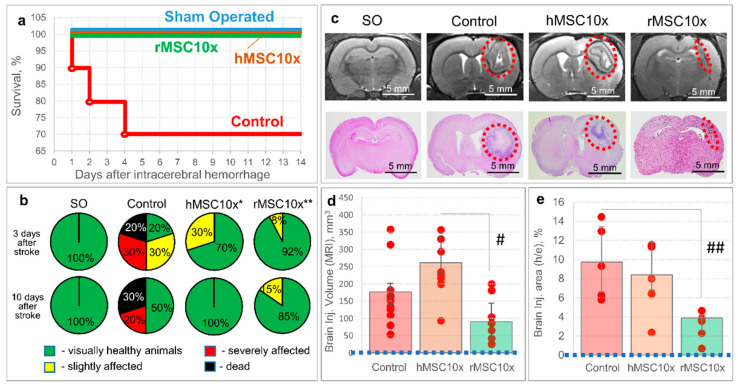
The results of in vivo studies of the neuroprotective activity of the 10 fold-concentrated rat (allogenic) MSC secretome (rMSC10x): (**a**) the dynamics of survival of the experimental animals; (**b**) neurological status of the experimental animals at days three and 10 after the intracerebral hemorrhage (SO—sham operated) [*—*p* = 0.006, n = 10, two-sided Fisher’s exact test; **—*p* = 0.0004, n = 18, two-sided Fisher’s exact test]; (**c**) samples of MR images and histological slices of the brains obtained from the experimental animals (the red dashed ovals restrict the injured loci); (**d**) results of the MRI examination of the brain at day 11 after the intracerebral hemorrhage [#—H (2, 21) = 11.033; *p* = 0.004; n ≥ 8; ANOVA on ranks]; (**e**) results of the histochemical examination of the brain at day 14 after the intracerebral hemorrhage [##—H (2, 12) = 7.580; *p* = 0.023; n = 5; ANOVA on ranks]. Data is presented as a median (25%; 75%).

As the CD68-specifically stained area in the hMSC10x group was significantly higher than in the other groups and correlated with the brain lesion volume according to MRI and histochemical studies, we believe that this negative effect is associated with the use of the xenogenic (human) MSC secretome. To verify our hypothesis, we studied the potential therapeutic effect of rMSC10x (an allogenic secretome) and compared its efficacy with that of hMSC10x (a xenogenic secretome), observing that the rMSC10x secretome provides 100% survival and an excellent neurological recovery of animals after the intracerebral hemorrhage, as with the hMSC10x secretome [*p* = 0.006 (n = 10) for hMSC10x vs. control; *p* = 0.0004 (n = 18) for rMSC10x vs. control; two-sided Fisher’s exact test] (Figure 6). According to the MRI study, the rMSC10x secretome effectively decreased the brain lesion volume, unlike hMSC10x: 90.2 (48.3; 144.3) mm^3^ for rMSC10x vs. 261.4 (232.0; 337.4) mm^3^ for hMSC10x [H (2, 21) = 11.033; *p* = 0.004; n ≥ 8; ANOVA on ranks]. Similar results were obtained within the histological study of the rat brains: the brain injury area in the rMSC10x group was significantly smaller (3.9% (2.6%; 4.2%)) than in the control group (9.8% (6.6%; 13.4%)) [H (2, 12) = 7.580; *p* = 0.023; n = 5; ANOVA on ranks] (Figure 6).

This lesion size shrinkage also correlated with the decreased CD68 immuno-staining in the brain slices of rMSC10x rats: 5.6% (1.1%; 7.7%) [H (5,48) = 16.689; *p* = 0.005 for rMSC10x vs. hMSC10x; n ≥ 9; ANOVA on ranks] (Figure 5a). CD163 staining in the brain slices of rMSC10x rats did not differ significantly from the other groups: 5.3% (2.2%; 5.5%) [H (5,42) = 10.589; *p* = 0.06; n ≥ 8; ANOVA on ranks] (Figure 5b).

### 3.3. MSC Secretome Supports the Survival of Neuroblastoma Cells in Glutamate–Induced Neurotoxic Conditions

To elucidate the possible cellular mechanisms that underlie the neurotrophic activity of MSC secretome, we studied its ability to stimulate the crucial stages of brain regeneration using in vitro models of cell survival under glutamate-induced neurotoxic conditions and neurite outgrowth.

**Figure 7:**
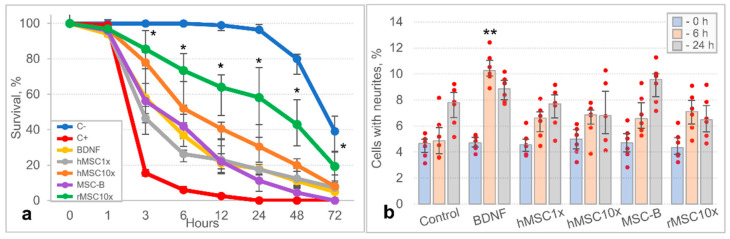
The results of in vitro studies of the neuroprotective activity of MSC secretomes (hMSC1x; MSC-B; hMSC10x; rMSC10x): (**a**) the dynamics of survival of SH-SY5Y neuroblastoma cells under glutamate-induced neurotoxicity conditions [*—H (5,66) < 45.430; *p* < 0.001 vs. control (C+); n = 12; ANOVA on ranks]; (**b**) neurite outgrowth in SH-SY5Y neuroblastoma cell culture [**—H (5,24) = 17.354; *p* = 0.004 vs. control; n = 5; ANOVA on ranks]. Data is presented as a median (25%; 75%).

We found that hMSC1x, hMSC10x, rMSC10x, and MSC-B secretomes support SH-SY5Y human neuroblastoma cell line survival under conditions of glutamate-induced neurotoxicity (Figure 7a, Figures S3 and S4). Thus, the number of living cells in the hMSC1x, hMSC10x, rMSC10x, and MSC-B groups was significantly higher in the 3–24 h after glutamate supplementation than in the positive control group “C+” without any secretome [H (5,66) < 45.430; *p* < 0.001 vs. control (C+); n = 12; ANOVA on ranks]. SH-SY5Y survival in the secretome groups was comparable to that observed in the positive control group BDNF. No significant differences in SH-SY5Y survival were observed between the MSC groups, with the exception of rMSC10x: its neuroprotective activity was significantly higher compared to the “C+” group and even compared to the hMSC1x, MSC-B, and hMSC10x groups at certain time points. The hMSC1x, hMSC10x, and rMSC10x secretomes supported SH-SY5Y survival up to 72 h under the neurotoxic conditions, with results of 7% (0%; 9%), 8% (4%; 10%), and 19% (11%; 27%) of living cells, respectively, whereas no living cells were observed after 24 h of the experiment in the “C+” group. In addition to glutamate-mediated toxicity, spontaneous death of SH-SY5Y cells was also observed, which increased significantly only in the first 24 h of the in vitro experiment and could be observed in the negative control group “C-” (serum-free medium without glutamate).

The study of the dynamics of SH-SY5Y neurite outgrowth during the 24 h experiment demonstrated the spontaneous neurite growth in all experimental groups. The hMSC1x, hMSC10x, MSC-B, and rMSC10x secretomes increased the neurite outgrowth compared to the control group within 6 h from the start of the experiment; nevertheless, the difference was not statistically significant. Only in the BDNF group was the neurite outgrowth significantly higher than the control group [H (5,24) = 17.354; *p* = 0.004 vs. control; n = 5; ANOVA on ranks] (Figure 7b and Figure S5).

## 4. Discussion

Intracerebral hemorrhage is a severe condition, comprising a number of pathological phenomena (neuronal death, damage to nerve fibers, inflammation, ischemia, fibrosis, etc.). Accumulated data suggests that MSCs can diminish and revert these pathological processes due to the production of an abundance of protein and non-protein bioactive compounds [3,5,11,12,13]. Our data, together with published data, show that the MSC secretome contains neurotrophins (BDNF, GDNF, NT3, etc.), growth factors (VEGF, HGF, bFGF, etc.), and microRNAs with neurotrophic activity [28,29]. It has been shown that MSCs can stimulate regeneration processes in a wide variety of tissues and, particularly, in nerve tissue [5,6,7,8,9,10].

Combining these two facts, we hypothesized that cell secretomes may alleviate the consequences of intracerebral hemorrhage and accelerate brain regeneration via the protection of neural cells, suppression of inflammation, and removal of fibrin deposits. Such complex effects of the cell secretome can be provided only by a combination of protein and non-protein molecules. However, some of these may have opposite effects [30,31].

Here, we tried to assess the potential effects of the MSC secretome for neuroprotection and brain recovery stimulation after intracerebral hemorrhage. In a model of intracerebral hemorrhage, growth factors with neurotrophic activity, which are a part of the secretome, could provide immediate neuroprotection to damaged neurons via the activation of protective ras/erk and PI3K cascades, as well as blocking proapoptotic Bcl-2 family proteins (Bax, Bak) [32,33]. Recent work has demonstrated the importance of BDNF secreted by MSC in brain neuroprotection after intraventricular hemorrhage in newborn rats [34].

The vesicular fraction of the secretome also influences the survival and regeneration of neural cells via the delivery of a wide range of proteins, messenger and regulatory RNAs, and even mitochondria, thereby changing the response of target cells to damage [35,36]. Several studies have shown that vesicles have tropism to certain types of cells [37,38]. Although the mechanism of this phenomenon is not well understood, it is believed that it is possible to control microvesicle tropism, thereby regulating the “behavior” of precise cell populations.

In our pilot experiment, the hMSC1x secretome did not provide any significant effect on survival or the alleviation of neurological deficits in experimental animals compared to the control groups. However, a visual observation of animals from the hMSC1x group showed that some of them that were in a critical condition after the intracerebral hemorrhage survived and demonstrated a rapid recovery. It should be noted that no significant differences in survival and severity of neurological deficit were observed between the positive (BDNF) and negative (Control) control groups in this experiment. We believe that this can be explained by the severity of the condition (intracerebral hemorrhage) and the insufficiency of action of a single molecule (BDNF), even though it has a strong proven neuroprotective effect.

Intriguing results were obtained when studying the cognitive abilities of rats during the pilot experiment, with animals from the hMSC1x group showing a tendency towards better retention of long-term memory at 10 days after the intracerebral hemorrhage: 100% (94.4%; 100%) versus 79.2% (12.2%; 100%) in the Control group (n ≥ 8) and 100% (38.9%; 100%) in the group of Sham-operated animals.

The totality of the results obtained in the pilot experiment allowed us to assume that the hMSC1x secretome has neurotrophic activity, but its low strength may be due to the low concentration of its components. Meanwhile, the observed delayed effect of the hMSC1x secretome on rat cognitive function is likely caused by the predominant action of its vesicular fraction, which regulates the “behavior” of neural cells close to the injury site by changing their transcriptional program [39].

To assess the possibility of the practical application of the MSC secretome, we decided to investigate the neurotrophic activity of the concentrated (hMSC10x) and combined (MSC-B) human MSC secretomes. We hypothesized that MSC-B could combine the immediate protective effect of neurotrophins (hBDNF) and the delayed pro-regenerative effect of cellular microvesicles from the MSC secretome.

According to the data of our extended study, concentrated (hMSC10x) and combined (MSC-B) secretomes decreased the mortality of the experimental animals after the intracerebral hemorrhage and improved their neurological status compared to the control and hMSC1x groups. However, the results of the MRI study showed a significant increment in the volume of brain lesions in the hMSC10x group compared to the BDNF, hMSC1x, and MSC-B groups, and even compared to the control group. Thus, despite an increase in survival of animals and an improvement in their neurological state, hMSC10x and MSC-B secretomes did not decrease the lesion focus, and there is a negative prognosis of the outcome for an intracerebral hemorrhage in the deferred period. The mechanism of this phenomenon is not well understood, especially since, according to the in vitro testing, hMSC10x and MSC-B secretomes stimulate the survival of SH-SY5Y cells under glutamate-induced neurotoxic conditions significantly better than BDNF or Control for at least 24 h.

Perhaps such differences in the action of hMSC10x and MSC-B secretomes in vivo and in vitro are associated with the complexity of the cellular composition in the hemorrhage focus: the presence of macro- and microglial cells, immune and vessel-belonging cells, in addition to neurons. This assumption was confirmed by the results of immunohistochemical staining of brain slices. In the hMSC10x and MSC-B groups, there is a greater intensity of CD68-specific staining (a marker of pro-inflammatory phagocytic and autophagocytic microglia [40]), and a decrease in staining for markers of anti-inflammatory hemoglobin-activated macrophages CD163 [41]. On the one hand, activated microglia cells promote early phagocytosis of blood components, which reduces its toxic effect [42,43]; on the other hand, hyperactivation of microglial cells increases the attraction of immune cells, which leads to expansion of the lesion area [44]. Perhaps the bigger lesion area in the hMSC10x and MSC-B groups is caused by hyperactivation of microglial cells by the vesicular fraction of the human MSC secretome, as vesicles carry species-specific MHC molecules [45,46]. These works demonstrate that extracellular vesicles can activate residual immune cells, especially if they are xenogeneic.

Combining the fact that administration of a xenogeneic MSC secretome increases the volume of the brain injury and increases the intensity of CD68-specific staining in the brains of experimental animals, we supposed that an allogeneic MSC secretome would be more effective. We studied the efficacy of the rat MSC secretome (rMSC10x) and found that it provided excellent survival and neurological outcome of experimental rats, which correlated with the shrinking of the lesion site and an approximately normal CD68-specific staining of the brain slices.

Interestingly, our preliminary results [20] and the published data [40,44] show that phagocytic activity and recruitment of immune cells are different processes (or degrees of activation) in microglia and can be controlled by adding or excluding individual components of the secretome. The study of the contribution of individual components of the MSC secretome to neuroprotection, neuroinflammation, and regeneration requires additional research.

The observed neurotrophic effect is specific for MSCs and their secretome. To prove the high therapeutic potential and exclusiveness of the MSC secretome, we previously demonstrated that it alleviates and reverts fibrosis in a model of pulmonary fibrosis [29]. Our unpublished data also confirmed that the HEK293 (human embryonic kidney) secretome does not provide such prominent survival, neurological outcome, and lesion shrinkage as the MSC secretome.

From the point of view of practical application, the proposed approach is unrealistic, as it requires the prompt administration of the cell secretome directly into the hemorrhage locus. However, as a proof-of-concept, it demonstrates the fundamental possibility of using a cell secretome for the treatment of intracerebral hemorrhage, especially after establishing the contribution of each secretome component. There is a high probability that the problem of delivery of trophic factors or certain secretome components across the blood-brain barrier and into the penumbra area will be resolved in the near future [47]. Furthermore, we plan to clarify the mode of action of the MSC secretome in order to determine its effects depending on the time and route of administration and to study the postponed effects of such a treatment.

## 5. Conclusions

As a result of this study, we found that the negative consequences of intracerebral hemorrhage (death, neurological deficits) can be largely prevented and mitigated by direct injection of MSC secretome into the focus of the hemorrhage. Depending on its composition, the secretome can have an immediate or delayed effect, and can also affect individual stages or cell-participants of the pathogenesis of the intracerebral hematoma.

According to the totality of tests, the combined secretome MSC-B (hMSC1x + BDNF) and, especially, the 10-fold allogeneic MSC secretome (rMSC10x) have the most impressive therapeutic activity. The hMSC10x secretome also turns out to be neurotrophic but, according to our MRI data, leads to an increase of the lesion focus. The mechanism of this counter-intuitive effect of the human MSC secretome and the key components responsible for neuroprotection and neuroregeneration remain to be determined.

## Data Availability

Data is available on request.

## Supplementary Materials

The following are available online at https://www.mdpi.com/article/10.3390/pharmaceutics13122031/s1, Figure S1. Histological signs of brain tissue damage (hematoxylin-eosin): (a,b) a necrosis focus restrained by neutrophil infiltration (NI) and a glial scar (GS); (c) signs of blood stasis and leukocyte migration through the capillary wall; Figure S2. Histological examination of brain slices. Perls stain reveals hemosiderin deposits (blue grains). Nissl stain reflects the functional state of neurons in the penumbra: red arrows show alive though hypoxic neurons, black arrows show dead neurons (neuron shadows); Figure S3. Samples of images (raw data) obtained during the study of neuroprotective activity of MSC secretomes in the model of in vitro glutamate-induced neurotoxicity. Green stain marks dead cells (apoptosis); Figure S4. Human and rat MSC secretomes support the survival of SH-SY5Y neuroblastoma cells under glutamate-induced neurotoxic conditions (scatter plots); Figure S5. Samples of images (raw data) obtained during the study of the ability of MSC secretomes to stimulate neuritogenesis.

## Abbreviations

ANOVA	Analysis of Variance
BDNF	Brain-Derived Neurotrophic Factor
bFGF	basic Fibroblast Growth Factor
DAPI	4′,6-diamidino-2-phenylindole
DMEM	Dulbecco’s Modified Eagle Medium
GDNF	Glial cell-Derived Neurotrophic Factor
GS	Glial scar
HGF	Hepatocyte Growth Factor
MRI	Magnetic Resonance Imaging
MSCs	Multipotent Mesenchymal Mtromal Cells
NI	Neutrophil infiltration
NT3	Neurotrophin-3
PI3K	Phosphoinositide 3-kinase
RNA	Ribonucleic Acid
SO	Sham Operated
VEGF	Vascular Endothelial Growth Factor
