# Supplementary material for: MSC Secretome as a Promising Tool for Neuroprotection and Neuroregeneration in a Model of Intracerebral Hemorrhage
Source: Pharmaceutics. 2021 Nov 29;13(12):2031. doi: 10.3390/pharmaceutics13122031 (PMC8707464; doi:10.3390/pharmaceutics13122031)
Supplement: Supplementary file 1 [file pharmaceutics-13-02031-s001.zip › pharmaceutics-1422669-SI.pdf]

pharmaceutics

Supplementary Materials: MSC Secretome as a Promising Tool for Neuroprotection and Neuroregeneration in a Model of Intracerebral Hemorrhage

Maxim Karagyaur *, Stalik Dzhauari, Nataliya Basalova, Natalia Aleksandrushkina, Georgy Sagaradze, Natalia Danilova, Pavel Malkov, Vladimir Popov, Maria Skryabina, Anastasia Efimenko and Vsevolod Tkachuk

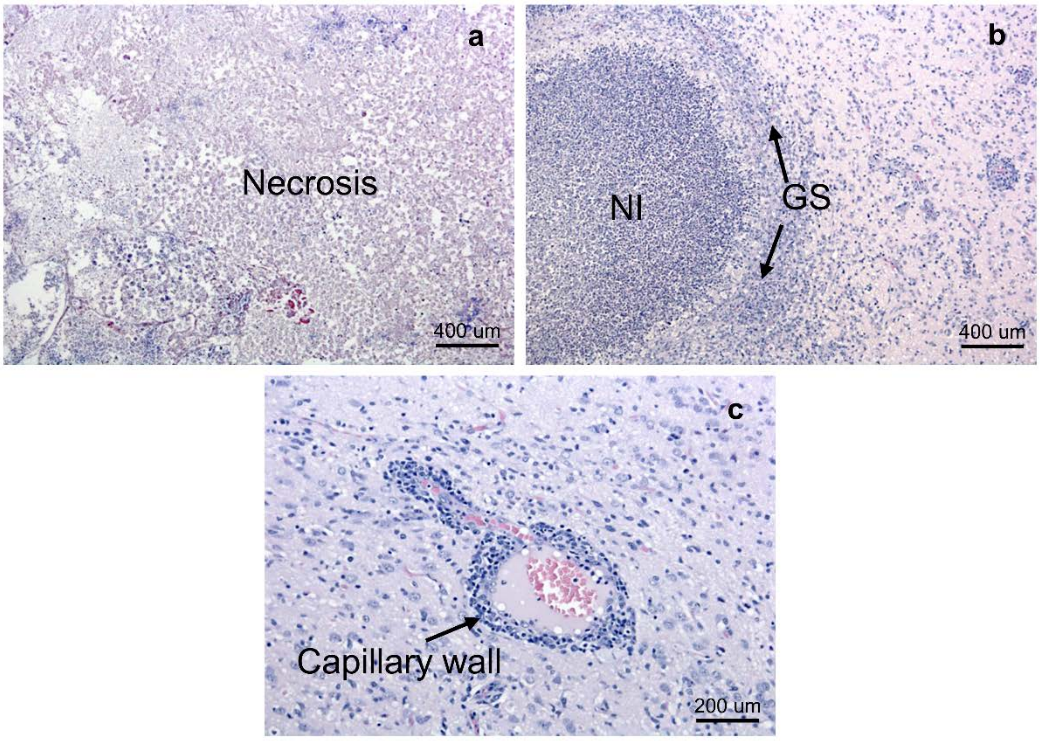

Figure S1. Histological signs of brain tissue damage (hematoxylin-eosin): (**a**,**b**) a necrosis focus restrained by neutrophil infiltration (NI) and a glial scar (GS); (**c**) signs of blood stasis and leukocyte migration through the capillary wall.

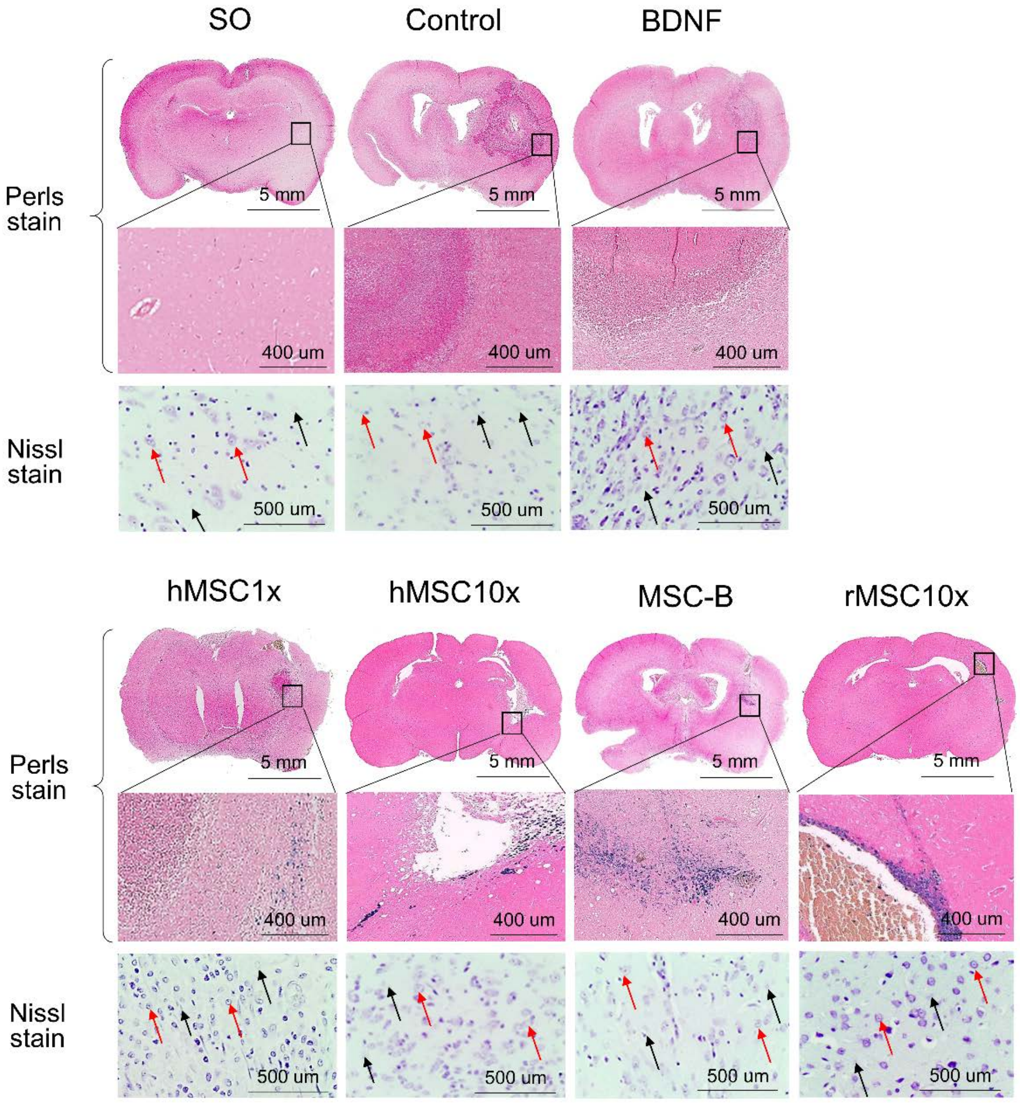

Figure S2. Histological examination of brain slices. Perls stain reveals hemosiderin deposits (blue grains). Nissl stain reflects the functional state of neurons in the penumbra: red arrows show alive though hypoxic neurons; black arrows show dead neurons (neuron shadows).

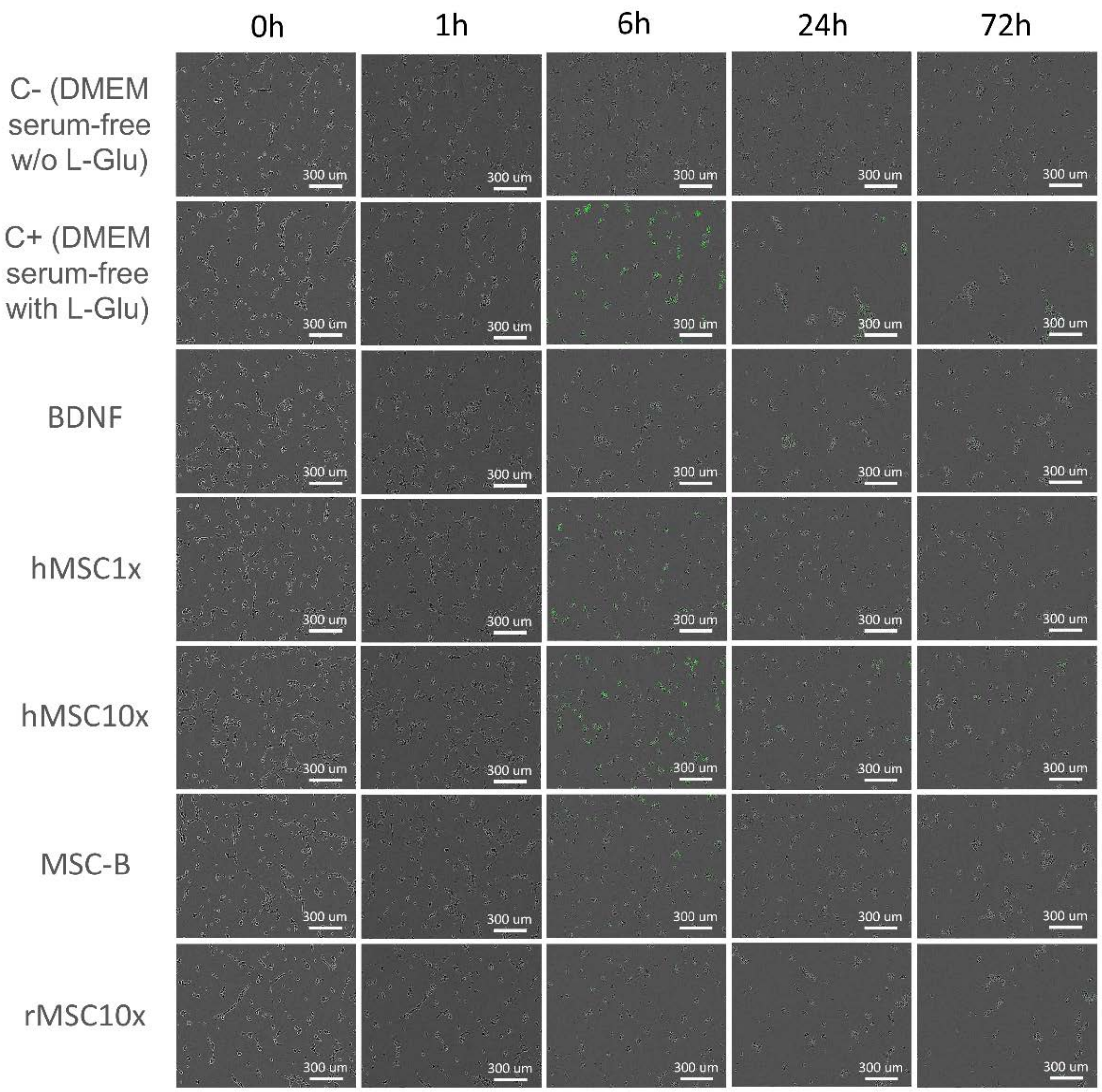

Figure S3. Samples of images (raw data) obtained during the study of neuroprotective activity of MSC secretomes in the model of *in vitro* glutamate-induced neurotoxicity. Green stain marks dead cells (apoptosis).

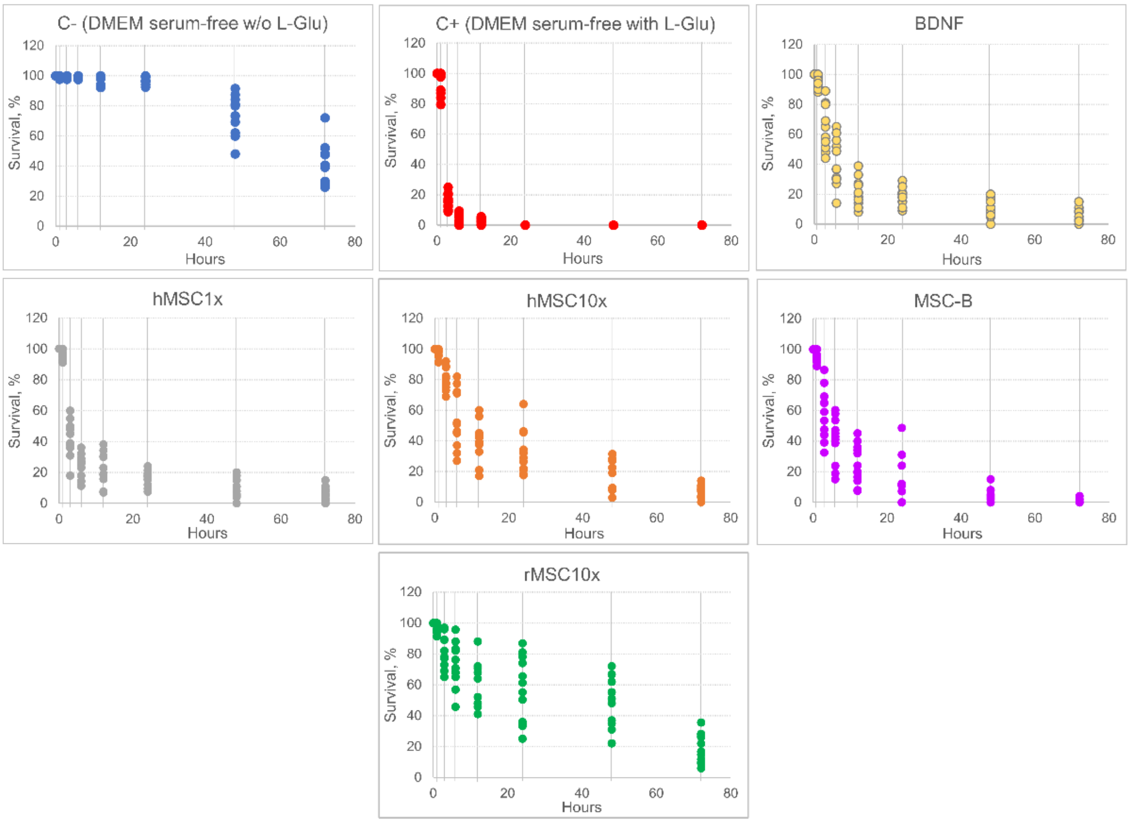

Figure S4. Human and rat MSC secretomes support the survival of SH-SY5Y neuroblastoma cells under glutamate-induced neurotoxic conditions (scatter plots).

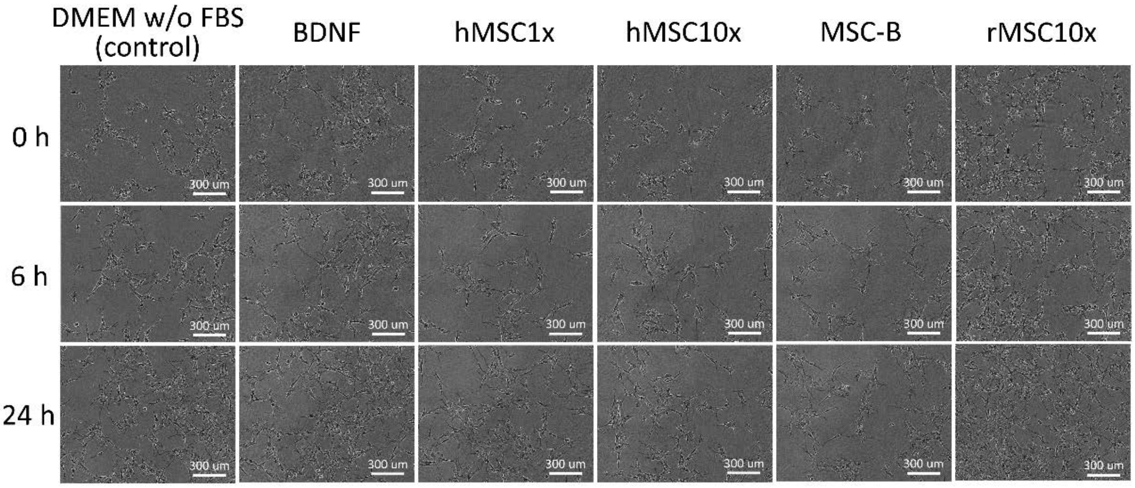

Figure S5. Samples of images (raw data) obtained during the study of the ability of MSC secretomes to stimulate neuritogenesis.
